# Combinatory Effect of Nitroxoline and Gentamicin in the Control of Uropathogenic Enterococci Infections

**DOI:** 10.3390/antibiotics13090829

**Published:** 2024-09-01

**Authors:** Davorka Repac Antić, Bruno Kovač, Marko Kolenc, Irena Brčić Karačonji, Ivana Gobin, Mirna Petković Didović

**Affiliations:** 1 Department of Microbiology and Parasitology, Faculty of Medicine, University of Rijeka, 51000 Rijeka, Croatia; davorkaar@medri.uniri.hr (D.R.A.); ivana.gobin@medri.uniri.hr (I.G.); 2 Department of Clinical Microbiology, Clinical Hospital Center Rijeka, 51000 Rijeka, Croatia; 3 Chair of Buildings and Constructional Complexes, Faculty of Civil and Geodetic Engineering, University of Ljubljana, 1000 Ljubljana, Slovenia; bruno.kovac@fgg.uni-lj.si; 4 Institute of Microbiology and Immunology, Faculty of Medicine, University of Ljubljana, 1000 Ljubljana, Slovenia; marko.kolenc@mf.uni-lj.si; 5 Division of Toxicology, Institute for Medical Research and Occupational Health, 10000 Zagreb, Croatia; ibrcic@imi.hr; 6 Department of Basic Medical Sciences, Faculty of Health Studies, University of Rijeka, 51000 Rijeka, Croatia; 7 Department of Medical Chemistry, Biochemistry and Clinical Chemistry, Faculty of Medicine, University of Rijeka, 51000 Rijeka, Croatia

**Keywords:** synergism, enterococci, *E. faecalis*, antiadhesion, proteome, antimicrobial resistance

## Abstract

*Enterococcus faecalis*, responsible for a majority of human and nosocomial enterococcal infections, is intrinsically resistant to aminoglycoside antibiotics (such as gentamicin, GEN), which must be used in a combined therapy to be effective. Nitroxoline (NTX) is an old antibiotic, underused for decades, but rediscovered now in an era of growing antibiotic resistance. In this in vitro study, the types of interactions between NTX and GEN on 29 *E. faecalis* strains were analyzed with an aim to find synergistic antimicrobial and antiadhesion combinations. Transmission electron microscopy (TEM) and matrix-assisted laser desorption/ionization time-of-flight mass spectrometry (MALDI-TOF MS) were used to analyze changes in cell morphology and bacterial proteome after monotreatments and combined treatments. The results showed the synergistic effect for six combinations on eight strains, including the ATCC29212, and an additive effect for most strains. Combinations causing a complete inhibition of adhesion were established. Cell membrane integrity was affected by NTX, while combined NTX/GEN treatment caused dramatic changes in cell morphology. Upregulation of the expression of many proteins was established, with some emerging only after combined treatment. The results strongly imply that NTX has the potential for use in combined therapy with GEN against enterococci and it could further provide a substantial contribution to an ongoing fight against antimicrobial resistance and nosocomial infections.

## 1. Introduction

Enterococci are Gram-positive bacteria, widely distributed in nature, in the soil, in the human digestive system, female genital tract, and oral cavity, and one of the leading causes of nosocomial infections [1]. For humans, the two most important species, *Enterococcus faecalis* and *Enterococcus faecium*, are known as opportunistic pathogens, causing endocarditis, septicemia, abdominal infections, and are identified as a major cause of urinary tract infections and bloodstream in intensive care units [2,3,4]. *E faecalis* is responsible for 80–90% of human enterococcal infections [5]. Enterococci are intrinsically resistant to low levels of aminoglycoside antibiotics (such as gentamicin, GEN) due to inefficient active transport across the cytoplasmic membrane [6]. Thus, aminoglycosides alone are considered inactive in the treatment of enterococcal infections and are usually combined with inhibitors of cell wall synthesis (such as ampicillin) which facilitate their uptake [7,8]. Enterococci are also notorious for their ability to form biofilms on urinary, gastrostomy, and intravascular catheters, which further increases their intrinsic resistance to antibiotics [3,9,10,11,12,13].

Nitroxoline (5-nitro-8-hydroxyquinoline, NTX) is an old antibiotic, in use from the beginning of the 20th century [14]. With the emergence of modern antibiotics, it has fallen out of wide use due to underestimation of its antimicrobial activities and is kept in use only in Eastern European countries and Germany [15,16,17]. With the proliferation of antibiotic resistance due to the overuse of typical antimicrobials, NTX underutilization appears to be a showcase for the “advantage of backwardness” concept. In the last few years, studies have demonstrated its antifungal [18], antiparasitic (among 2,177 clinically approved compounds, NTX was identified as the most promising candidate for the treatment of life-threatening *Balamuthia mandrillaris* infections [19]), antiviral [20], antibiotic [21,22], antiangiogenic, and anticancer activity [23]. However, it is still best known as an effective uroantiseptic that shows no pronounced toxic side effects [24,25]. Chemically, it is a heterocyclic aromatic compound from the family of 8-hydroxyquinoline derivatives (8-HQ), a group of compounds with an extremely wide spectrum of pharmacological [26,27,28] and non-pharmacological effects [21,24]. The 8-HQ core of its structure belongs to the so-called “privileged structures” due to its versatile binding properties [29]. NTX does not belong to any known group of antibiotics, due to its unique mechanism of action that rests almost exclusively on its chelating properties [21,30,31,32]. Chelation is also responsible for the unique ability of NTX to inhibit bacterial adhesion and biofilm formation [33,34,35,36,37].

Our preliminary results on NTX’s antiadhesion and antibiofilm action against *E. faecalis* [30] led us to further study its actions against this nosocomial pathogen. The main focus of this research was to establish a potential synergistic or additive effect of NTX and aminoglycosidic antibiotic GEN against *E. faecalis* and try to elucidate the origin of the effect. For that purpose, the susceptibility profiles of 29 *E. faecalis* strains were first measured for both antimicrobials and compared to the effect of the combined treatment with various concentration combinations. Then, the antiadhesion actions of monotreatments were examined for the selected strains and compared to the combined treatments. Finally, the matrix-assisted laser desorption/ionization time-of-flight mass spectrometry (MALDI-TOF MS) spectra were recorded to analyze the effect of monotreatments and combined treatment on *E. faecalis* proteome in order to gain some insight into the mechanism of the combined action.

## 2. Results

### 2.1. Susceptibility Profile of *E. faecalis* Strains for GEN and NTX

**Figure 1:**
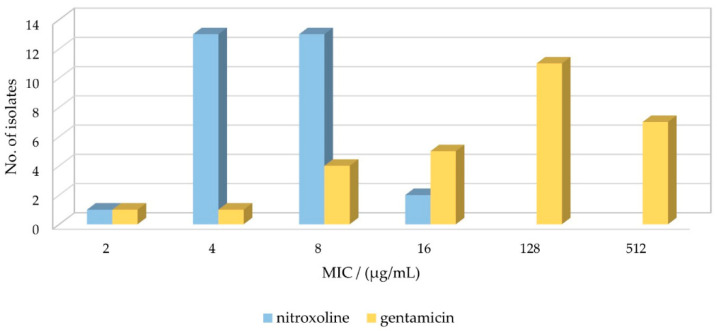
Susceptibility of *Enterococcus faecalis* isolates to nitroxoline and gentamicin. MIC–minimum inhibitory concentration.

As a first step in establishing the effect of GEN and NTX individual antibacterial activity, the minimum inhibitory concentrations (MIC) were measured for 29 *E. faecalis* strains. As shown in Table 1 and Figure 1, the MIC values of GEN ranged from 2 µg/mL up to 512 µg/mL. To assess the activity, the values should be compared to the clinical breakpoints determined by the European Committee on Antimicrobial Susceptibility Testing (EUCAST). However, GEN belongs to aminoglycoside antibiotics, a group to which enterococci are resistant when used in monotherapy, thus there are no available data in the newest EUCAST report [38]. Similar to other studies [22], we used the clinical breakpoints of *Escherichia coli*, which is for GEN reported as 2 µg/mL for infections originating from the urinary tract [38]. Based on this value, only 1 out of 29 strains was found to be gentamicin susceptible (MICs ≤ 2 μg/mL), while 28/29 were gentamicin resistant (MICs > 2 μg/mL). Note that EUCAST implemented new definitions of susceptibility testing categories S (Susceptible, standard dosing regimen), R (Resistant), and I (Susceptible, increased exposure) in 2019 [39]. 

Regarding the MIC values of NTX for 29 *E. faecalis* isolates, for 90% of them, the MICs of 4 µg/mL (45%, 13/29) and 8 µg/mL (45%, 13/29) were obtained. The MIC was lower (2 µg/mL) for only one strain, and higher (16 µg/mL) for two strains. The distribution and comparison to GEN MIC values are shown in Figure 1. There is no data in the EUCAST report regarding NTX clinical breakpoints for *E. faecalis* due to insufficient evidence, so again we used *E. coli* breakpoints, which were determined as 16 μg/mL for uncomplicated urinary tract infections [38]. Based on this value, 100% of the tested isolates were susceptible to NTX. The results are in good agreement with those obtained by Deschner et al., where MICs ≤ 16 µg/mL were measured for *E. faecalis* ATCC29212 and MHH83300 strains [22]. Cherdtrakulkiat et al. [40] reported for ATCC29212 (and ATCC33186) strain the MIC value of 42.07 µmol/L, which translates to 8 µg/mL, matching ours for the same strain. Sobke et al. determined MICs for 139 *E. faecalis* strains (unspecified), ranging from 2 to 64 µg/mL, with Gauss distribution peaking at 8 µg/mL [41].

The minimum bactericidal concentrations (MBC) and minimum antiadhesion concentrations (MAC) were also determined for NTX (Table 1). The MBCs of 32 µg/mL were obtained for 83% of the isolates and >64 µg/mL for the rest. These values are on average three dilutions higher than the respective MICs, which is in line with NTX’s primarily bacteriostatic nature [41]. MAC values were, as a rule, one dilution lower than the respective MICs. This result indicates that the subinhibitory concentrations of NTX, while not sufficient to inhibit the growth of microorganisms, could have affected their ability to adhere to the surface through the modification of the physico-chemical properties of bacterial cells [42,43,44].

Overall, the results indicated higher tolerance of most *E. faecalis* strains to GEN than to NTX, which complies with the known high-level resistance of *E. faecalis* to aminoglycosides [12,45,46].

### 2.2. Synergism Testing of Selected Concentrations of Nitroxoline and Gentamicin on Uropathogenic E. faecalis Strains Using the Checkerboard Assay 

To analyze the effect of NTX and GEN combined action in comparison to monotreatments, all *E. faecalis* strains listed in Table 1 were exposed to various combinations of NTX and GEN subinhibitory concentrations. New MIC values were determined and used to calculate Fractional Inhibitory Concentration (FIC) using the following formula:

FIC(A) = MIC(A with B)/MIC(A)(1)

FIC(B) = MIC(B with A)/MIC(B)(2)

FIC values were then used to calculate the Fractional Inhibitory Concentration Index (FICi):

FICi = FIC(A) + FIC(B) = [MIC(A with B)/MIC(A)] + [MIC(B with A)/MIC(B)].(3)

With FICi values determined, the interaction of NTX and GEN in various combinations of subinhibitory concentrations could be categorized into the following categories [47,48]:

- Synergistic (FICi ≤ 0.5);
- Additive (0.5 > FICi ≤ 1.0);
- Indifferent (1.0 > FICi ≤ 4);
- Antagonistic (FICi > 4).

This method provides a clear distinction between synergistic and additive effects, which is frequently not made [49]. The checkerboard assay results are summarized in Table 2 and Table 3. The synergistic effect was determined in three NTX/GEN combinations for Group 8 and another three for Group 2 *E. faecalis* strains (Table 2). The two dilutions with lower concentrations of NTX and two to four dilutions with lower concentrations of GEN compared to monotreatment values, were sufficient to inhibit the growth of those strains. This important result demonstrates a powerful improvement of activity when GEN and NTX are used in a combined treatment with selected concentrations. 

The additive effect was the dominant interaction for all 10 groups of *E. faecalis* strains and at least three concentration combinations were determined (Table 3). The combinations were comprised of, on average, one dilution lower concentration of NTX, and from one down to six dilutions of lower concentrations of GEN compared to monotreatment. Note that the additivity is not based on the simple addition of effects of the two drugs, but rests on the dose equivalence concept, meaning that one component can be replaced with the other of the equivalent effect without influencing the overall antimicrobial efficacy [49]. 

The results reveal strain-specific synergistic interactions of NTX and GEN and broad additive interaction for 29 *E. faecalis* strains used in this study. Based on the obtained data, it appears that NTX could act as an efficient adjuvant for GEN in fighting *E. faecalis* infections, at least for some strains, especially in comparison with rare other studies. For instance, the study of Thieme et al. [49] did not determine the synergistic effect of the ampicillin/gentamicin combination for any of the examined *E. faecalis* strains. Jacobs et al. found that no synergism could be demonstrated with sulphamethizole for urinary tract infections [50].

As aforementioned, the results showed that the interactions are strain-specific, which complies with other studies demonstrating that the phenotypic and genetic characteristics of a strain are highly influenced by external conditions (e.g. environment conditions such as temperature, pH, nutrient availability, the presence of other microorganisms, the source of isolation, etc.) [13,51,52,53,54]. For instance, Abril et al. have recently shown how the number of peptides connected to virulence factors markedly varies in enterococcus strains depending on the dairy source from which they were isolated [55]. 

### 2.3. Testing Inhibition of Biofilm Formation at Selected Concentrations of Nitroxoline and Gentamicin 

The biofilm-forming ability of *E. faecalis* is known as a major factor in its virulence and drug resistance [10,11,56,57,58]. Therefore, we wanted to analyze and confirm the synergistic/additive effect of NTX and GEN established by MIC measurements by testing the inhibition of biofilm production. Selected NTX/GEN combinations were applied on selected strains and the effect was compared to monotreatments. The combinations were selected based on the frequency of appearance in the “synergistic” and “additive” categories used in MIC measurements. ATCC29212 was chosen because it is a reference strain often used in biofilm production and adhesion studies [57,59,60,61]; it was also taken as a representative of Group 4, for which the synergistic NTX/GEN effect has been established (Table 2). Strain E55 was taken as a representative of Group 8, the second group with established synergy. E1 and E15 were chosen as the representatives of strains with proven additive effects (Table 3).

**Figure 2:**
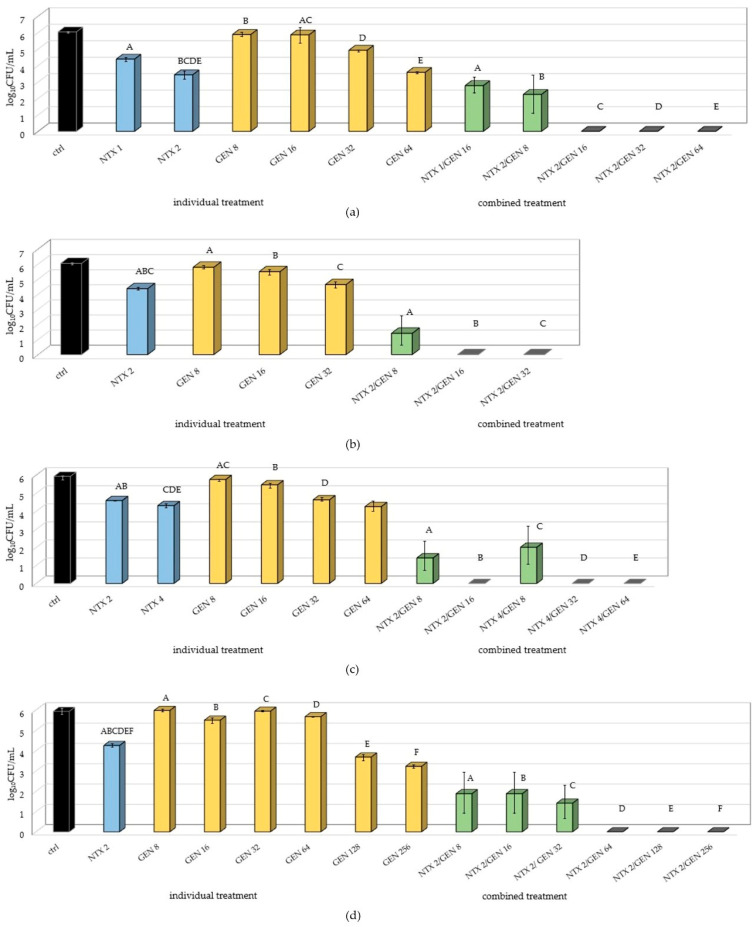
Number of adhered bacteria of (**a**) E15; (**b**) ATCC29212; (**c**) E55; and (**d**) E1 *Enterococcus faecalis* strains on polystyrene after monotreatments with nitroxoline (NTX), gentamicin (GEN), and combined treatment with selected concentrations. The result is presented as mean ± SD. Uppercase letters (A–F) mark statistically significant differences (*p* < 0.05) between combined treatment and individual treatments contained in the combined treatment. For example, the uppercase letter A in (**a**) marks a statistically significant difference between NTX1/GEN16 (combined treatment) and NTX1 and GEN16 (individual treatments). Also, uppercase letters BCDE above NTX2 mark a statistically significant difference between NTX2 and combined treatments NTX2/GEN8, NTX2/GEN16, NTX2/GEN32, and NTX2/GEN64.

The results (Figure 2) showed a significantly lower number of adhered bacteria when combined treatment was applied, indicating successful inhibition of biofilm formation for all applied NTX/GEN combinations. Complete inhibition was achieved in 11 out of 19 tested combinations. The statistically significant inhibition was accomplished for all four tested strains, but not to the same extent. The inhibition was combination-dependant and generally, the increase in GEN concentration for the fixed NTX concentration enhanced the inhibition. On the other hand, doubling the NTX concentration for the fixed GEN amount did not yield statistically significant differences (Figure 2a,c).

Besides successful inhibition of biofilm formation when NTX/GEN combinations were applied, the results also showed statistically significant differences between NTX and GEN monotreatments. In all the tested strains, GEN concentrations of 8 µg/mL and 16 µg/mL did not cause significant changes in the number of adhered bacteria compared to the control. For the E1 strain, it took as high as 128 µg/mL for the occurrence of inhibition. This result is in accordance with susceptibility profiles determined in Section 2.1 and arises from the intrinsic tolerance of *E. faecalis* to aminoglycosides [12,45,46]. However, NTX monotreatment significantly decreased the number of adhered bacteria when used in concentrations as low as 1–4 µg/mL. For the E15 strain, the 1 µg/mL concentration decreased the count by almost 2 log_10_CFU.

Even though GEN alone did not exhibit significant biofilm production inhibition, the combination with NTX caused a drastic effect for most concentration combinations, with a complete inhibition for some combinations in all tested strains.

### 2.4. The Effect of the NTX/GEN Combined Treatment on the Morphology of ATCC29212 Strain Cells

**Figure 3:**
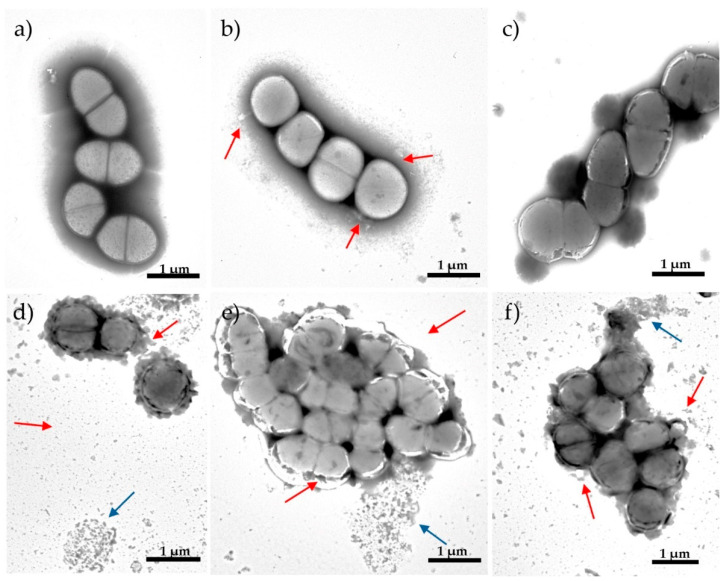
Transmission electron microscopy (TEM) micrographs showing morphological changes of *Enterococcus faecalis* ATCC29212 strain: (**a**) control; after 12 h of exposure to (**b**) nitroxoline (NTX) 8 μg/mL, (**c**) gentamicin (GEN) 8 μg/mL, (**d**) synergistic NTX/GEN combination 2/0.5 μg/mL, (**e**) additive NTX/GEN combination 2/4 μg/mL, and (**f**) additive NTX/GEN combination 4/0.5 μg/mL. Red arrows indicate cell wall disruption; blue arrows indicate cellular debris (decayed cells).

To gain some insight into the possible mechanism that led to the NTX/GEN combined effect implied by results in Section 2.2 and Section 2.3, morphological changes of bacterial cells caused by mono- and combined NTX/GEN treatments were examined, using ATCC29212 as the standard reference strain. Transmission electron microscopy (TEM) of the non-treated cells showed that the *E. faecalis* were mostly coccoid with an average diameter of 2.3–2.8 mm (Figure 3a). The cell membranes were intact and the presence of septum structure before complete cell separation was evident.

The images obtained using TEM showed various morphological changes in bacterial cells exposed to NTX or GEN, compared to untreated control bacteria. Exposure to MIC concentrations of NTX at 8 µg/mL and GEN at 8 µg/mL for 12 h led to certain morphological changes in bacterial cells. Lack of cell shape, absence of septum, and leakage of cellular material (Figure 3b) can be seen after treatment with NTX. Treatment with GEN did not cause visible damage to the cell wall but resulted in cytoplasm thickening and the presence of septation.

In contrast to the mono-treatment of MIC concentration, the subinhibitory concentration of combined NTX/GEN treatment caused dramatic changes in cellular morphology. After treatment with the synergistic combination of NTX/GEN (2/0.5 μg/mL), the remaining cells decreased in overall cell size because of degenerative changes to the cell wall, which included thickening, disruption, increased roughness, and the reduction in the amount of cytoplasm, which is related to the proper functioning of the cell membrane as a barrier (Figure 3d). The majority of the observed debris resulted from the decay of cells on copper grids.

After treatment with the additive combination of NTX/GEN similar morphological changes were observed. The cell cytoplasm was unevenly condensed, and there were significant gaps between the cytoplasmic membrane and the cell wall. Some cells appeared to have vacuolar degeneration and a loose uneven cytoplasmic matrix (Figure 3e,f).

### 2.5. MALDI-TOF MS Analysis of ATCC29212 Wild and Mutant Strains

**Figure 4:**
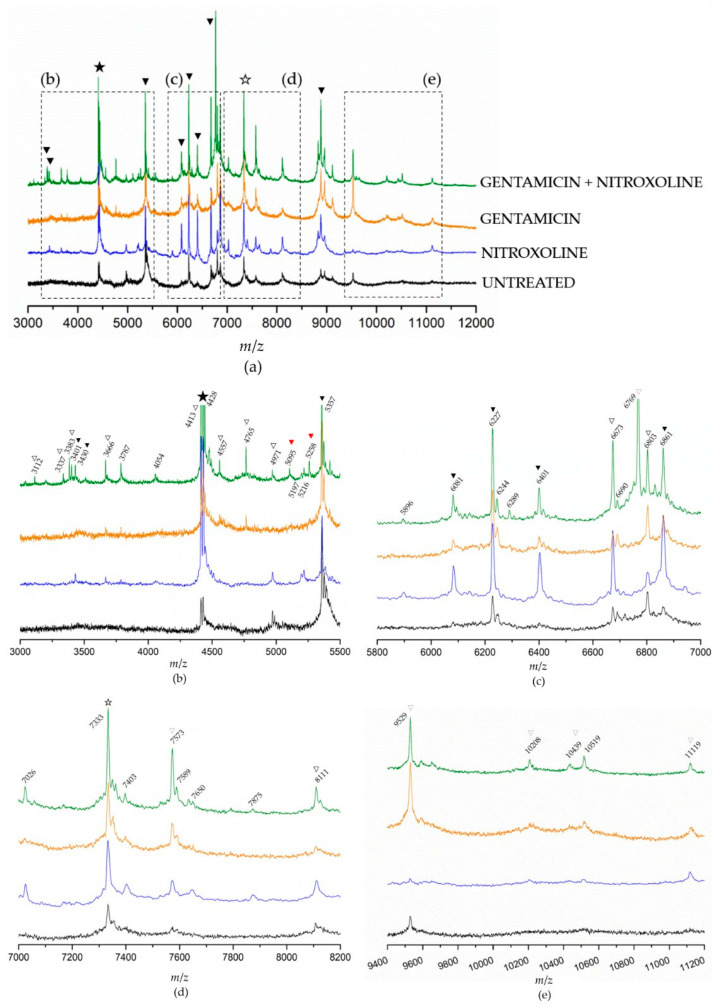
Matrix-assisted laser desorption/ionization time-of-flight mass spectrometry (MALDI-TOF MS) spectra of the *Enterococcus faecalis* control strain ATCC29212 without treatment, after monotreatments with nitroxoline and gentamicin, and after the combined treatment. The spectra are moved up the y-axis for easier comparison. Complete spectra are given in (**a**) and designated sections are zoomed in in images (**b**–**e**). The full and empty stars designate genus-specific and *E. faecalis*-specific biomarkers. Full black triangles designate species-characteristic peaks (found in *E. faecalis* strains, but also in the strains of other species); full red triangles designate peaks not found within the fingerprinting data of *E. faecalis* strains [62]; empty triangles designate peaks found only in certain *E. faecalis* strains.

To obtain additional information that could help elucidate NTX/GEN combined effect, we analyzed the differences in protein expression caused by the treatments of NTX and GEN individually and in combination. MALDI-TOF MS spectra of *E. faecalis* ATCC29212 mutant strains were recorded and compared to a wild (untreated) strain, as shown in Figure 4. Because the spectra contained an abundance of peaks in the range from 3 to 12 kDa (Figure 4a), they were sectioned into smaller regions to gain clearer insight (Figure 4b–e). First, a prominent peak at 4428 Da could be identified as a genus-specific biomarker (full star), found in MS spectra of all *Enterococci* strains [4,62,63]. Then, species-characteristic peaks (black triangles) were identified using proteomic fingerprinting data [62]. Eight such peaks could be identified in the shown spectra: 3401 Da, 3430 Da, 5357 Da, 6081 Da, 6227 Da, 6401 Da, 6861 Da, and 8883 Da. These peaks are species-characteristic because they have been found in MS spectra of all *E. faecalis* strains, but they were also found for some strains of *E. faecium* [62,63]. On the other hand, the 7333 Da peak (empty star) is characteristic exclusively for *E. faecalis*. Peaks labeled with an empty triangle are the ones found only in certain *E. faecalis* strains (if rarely, then the triangle is light grey) [4,62,63].

**Figure 5:**
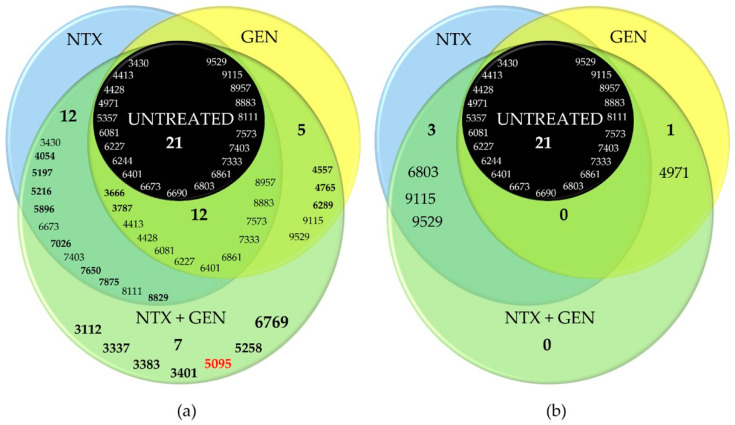
Venn diagrams of four tested *Enterococcus faecalis* ATCC29212 mutants (untreated; treated with nitroxoline, NTX; treated with gentamicin, GEN; treated with NTX/GEN combination) and their significantly (**a**) upregulated and (**b**) downregulated peaks. The four-digit numbers represent the *m*/*z* positions of peaks extracted from matrix-assisted laser desorption/ionization time-of-flight mass spectrometry (MALDI-TOF MS) spectra shown in Figure 4. Bolded values represent peaks not present in the untreated strain. Single- or double-digit numbers represent the total peak count in the particular category. Red number designates the peak found in *vanB*-positive isolate of *Enterococcus faecium*.

The intensity of all peaks varied depending on the treatment. Generally, it can be noticed that the intensities were the lowest in the spectrum of untreated bacteria, followed by GEN-treated and NTX-treated bacteria. Bacteria treated with the GEN-NTX combination yielded the spectrum containing the greatest number of peaks, with the highest intensities in most cases, indicating upregulation of the expression of most proteins as a general trend. The intensity of some peaks increased only in the NTX-treated mutant, others only in the GEN-treated mutant, while some increased in both. All upregulations from individual treatments were reflected in the combined treatment, without exception. In addition to those, the spectrum of the combined-treatment mutant contained several new peaks. To facilitate the analysis, these results were visualized using a Venn diagram (Figure 5). A total of 21 peaks could be identified in the wild-type proteome. Ten of them had increased intensities and two new peaks emerged (bolded in Figure 5a) unselectively, in both individual and the combined treatment. On top of this, NTX alone caused the increase of intensities of six peaks and the emergence of six additional ones. In contrast, GEN alone caused the increase of intensities of two peaks and the emergence of three additional ones. The combined treatment reflected those changes but also contained seven new peaks. Among those, the most prominent one was located at 6769 Da. Five of the new peaks (3112 Da, 3337 Da, 3383 Da, 3401 Da, and 6769 Da) were identified as species-characteristic (3401 Da) or present in the majority of *E. faecalis* strains, as labeled in Figure 4.

However, the peaks at 5095 Da and 5258 Da were not found within the fingerprinting data of *E. faecalis* strains [62]. The peak at 5095 Da draws special attention, because it has been found in the spectrum of *E. faecium* species, but only in the *vanB*-positive isolate [64]. *VanB*-positive designates the isolate that contains *vanB* gene cluster that renders the isolate vancomycin-resistant. In *E. faecalis*, *vanB* gene expression was studied on a V583 strain [65].

Note that the downregulation of protein expressions was markedly less pronounced compared to upregulation, as can be clearly seen from the Venn diagram in Figure 5b. The treatment with NTX and GEN individually caused the diminishing of intensities of only three and one peaks, respectively. In the combined treatment, no downregulation was recorded.

## 3. Discussion

The results of this study provide a strong indication of the NTX and GEN synergistic action against *E. faecalis* ATCC29212, certain other strains (Table 2), and additive action against the majority of the 29 examined strains (Table 3). The synergistic effect was also manifested as a successful inhibition of biofilm production for ATCC29212 and three other strains (Figure 2). TEM micrographs revealed severe cell morphology changes upon combined treatment (Figure 3). MALDI-TOF MS analysis revealed that NTX monotreatment caused upregulation of a higher number of protein expressions compared to GEN monotreatment (Figure 4 and Figure 5). Seven new peaks emerged in the spectrum of the combined-treated mutant, among which the peak characteristic for *vanB*-positive *E. faecium* isolate was also present. Downregulation of expression was markedly less pronounced, amounting to three downregulations by NTX, one by GEN, and none in the combined treatment.

While detailed identification of the proteome was outside the scope of this study, other useful information could be obtained. In MALDI-TOF MS spectra, the *m*/*z* range of 2–20 kDa encompasses mainly ribosomal proteins [66]. The upregulation of expression observed in the spectrum of NTX-treated mutants indicates certain (direct or indirect) interactions of NTX with many of those proteins, while the interactions with GEN were less pronounced. The proteins obtain various functions, including molecular functions, which act as cellular components or regulate biological processes. Zhang et al. [67] found that two distinct proteins, at *m*/*z* locations 10,283 and 11,278, were closely related to antimicrobial resistance (AMR). Upregulation of those proteins’ expression was deemed a reasonable response of bacterial cells when they are stimulated by antibiotics. Our results showed the upregulation of peaks at similar positions (10,208 Da and 11,119 Da, Figure 4e) in the spectra of both NTX-treated and GEN-treated mutants, which provides ground for speculation that these might be markers of proteins related to AMR in *E. faecalis*. The treatment with NTX/GEN combination yielded a peak at 5095 Da, which has been found in the *vanB*-positive isolates of a closely related *E. faecium* species [64]. This suggests that the combination of NTX and GEN stimulated the expression of this AMR-related protein, while monotreatments did not.

Alongside those three peaks, numerous other upregulations were observed, while downregulations were scarce. Namely, 21 peaks were counted in the spectrum of the untreated isolate, while 57 were observed in the spectrum after the combined treatment. In *E. faecalis*, 58 proteins are known to be involved in the inorganic ion transport and metabolism [2]. Since NTX’s mode of action rests primarily on its chelation ability [30], it is reasonable to assume that those proteins would also be upregulated by the presence of NTX. Indeed, it has been proven that genes responsible for iron acquisition are significantly upregulated in NTX-treated biofilm [33] and that there is a connection between NTX and iron or iron-sulphur cluster proteins [22]. In the same study, it was shown that NTX affects histidine kinase EnvZ, necessary for cell homeostasis and iron transport. Furthermore, Wagenlehner et al. [68] found that NTX in *E. coli* affects the number of adhesins, which indirectly implies that NTX interacts with proteins responsible for adhesins production. In *E. coli*, this was manifested as the inhibition of adhesion to human bladder epithelial cells. Our results showed a significant inhibition of adhesion of *E. faecalis* upon treatment with NTX. Additionally, in *E. faecalis*, the only protein that has been directly linked to biofilm formation is Enterococcal Surface Protein, Esp [2,3,69,70], thus it is reasonable to suspect that Esp was also affected by NTX. At the same time, GEN targets different cellular pathways, attacking the 30S ribosomal subunit and disrupting RNA formation, affecting bacterial protein synthesis. It is possible that the upregulation of expression of such a high number of proteins, in combination with the defense mechanism against GEN, might have caused cellular stress overload and pushed the bacteria over the tipping point.

Of course, GEN cannot bind to the 30S ribosomal subunit if it is unable to penetrate the cellular membrane and it is known that *E. faecalis* is relatively impermeable to aminoglycosides (which explains GEN’s high MIC values and low susceptibility profile of *E. faecalis* for GEN monotherapy, Section 2.1). This is the underlying reason why, for a successful antimicrobial action, GEN must be combined with ampicillin, a β-lactam antibiotic that targets the bacterial cell wall. Deschner et al. [22] have recently demonstrated that the resistance caused by NTX in *E. coli* is directly associated with bacteria fitness loss. It was found that NTX affects membrane porins OmpC and OmpF, and membrane-bound maltoporin LamB, which could explain our findings that NTX affected the cell membrane integrity of *E. faecalis* (Figure 3b). OmpF and LamB were found to be significantly downregulated by NTX, which can be correlated to three downregulations observed for NTX in this study (Figure 4b). It is worth mentioning that Bourlioux et al. [35] postulated 35 years ago that NTX acts at the outer membrane level of the bacterial cell wall by a chelating effect preferentially with Mg^2+^ than Ca^2+^. All this amounts to a viable explanation of NTX and GEN synergistic effect.

The results of this study also showed that NTX alone and in combination with GEN significantly reduces biofilm formation in vitro. Reduction in biofilm formation was obtained at sub-MIC values. The effect of NTX on biofilm is due to its chelating ability, although the detailed antibacterial mechanism of action has not yet been elucidated. Its action is generally considered to be indirect as it acts bacteriostatically by chelating cations essential for bacterial growth [71]. Iron serves as a cofactor in enzymes, electron-carrying proteins, RNA/DNA metabolism, and regulates biofilm formation. Due to the crucial role of iron, bacteria have had to evolve versatile iron acquisition systems, one of which is the secretion of siderophores (iron carriers), small molecules with a strong tendency to chelate iron [30,72], and for which NTX presents a direct competition. Additionally, it is well known that bacterial cells in biofilm communicate and coordinate their behavior through signal molecules, which is known as Quorum Sensing (QS) [73]. QS controls major virulence determinants in *E faecalis* [74]. One of the roles of QS is siderophore production, hence NTX might have acted as a QS inhibitor and in this way inhibited biofilm formation, alongside the aforementioned disruption of adhesins production [68,75]. Combined, NTX might have an impact on the early stages of biofilm production, as well as during proliferation and maturation [73]. These antiadhesion and antibiofilm NTX properties gain additional significance in light of information that over 65% of nosocomial infections originate from biofilm-related infections, while the use of medical implants is constantly increasing [3].

## 4. Materials and Methods

### 4.1. Strains, Culture Conditions, and Chemicals

In this research, 29 strains of *E. faecalis*, collected from clinical urine samples from patients with proven urinary tract infection (UTI) during routine work at the Clinical Microbiology Institute of Clinical Hospital Center Rijeka in Croatia, were used. The choice of 29 strains–out of around 100 that were isolated and analyzed in the course of our previous research [76]–was made based on phenotypic characterization, antimicrobial susceptibility, and biofilm-producing ability. HLAR (High-Level Aminoglycoside Resistance) screening to GEN was performed in order to determine strains with high- and low-level aminoglycoside resistance. All analyzed strains were ampicillin and vancomycin susceptible. Additionally, a standardized strain from the American Type Culture Collection (ATCC), *E. faecalis* ATCC29212, was also included. Before conducting the tests, the strains were stored in 10% glycerol at –80 °C. The bacteria were cultured on Mueller Hinton (MH) agar for 24 h at 35 ± 2 °C. The basic suspension of GEN and NTX was prepared in the medium recommended by the manufacturer and stored in aliquots at –20 °C until use [77]. NTX was dissolved in dimethyl sulfoxide (DMSO) (Sigma, Taufkirchen, Germany), while GEN was dissolved in sterile distilled water.

### 4.2. Determination of Minimum Inhibitory Concentrations of Selected Uropathogenic Enterococci

Antimicrobial susceptibility of clinical isolates of *E. faecalis* and the control strain (*E. faecalis* ATCC29212) was assessed using the microdilution method. The results were interpreted according to the valid EUCAST standard [38]. In 96-well microtiter plates (Vacutest Kima s.r.l., Arzergrande, Italy), serial double dilutions of the tested compounds were made in concentrations ranging from 0.25 µg/mL to 256 µg/mL for NTX and GEN. Bacterial suspension (1 × 10^6^ CFU/mL) was added to each well. Three wells were used as sterility controls, and three as growth controls. After 24-hour incubation at 37 °C, the plates were visually examined. The lowest concentration that inhibited the visible growth of the bacteria was defined as the MIC. MBC was determined by inoculating the test dilutions from each well without visible growth on MH agar and incubated for 24 h at 37 °C. The MBC was defined as the lowest concentration of antibiotics that killed ≥ 99% of the bacteria. The results were expressed in µg/mL. MAC was determined as the minimum dose of NTX and GEN that completely inhibited *Enterococcus* adhesion to polystyrene. Serial double dilutions of the tested compounds with bacteria suspension were made as previously described for MIC detection. After 24 h of incubation at 37 °C, the supernatant containing non-adherent bacterial cells was removed, the microtiter plates were washed twice with sterile phosphate-buffered saline (PBS) and sonicated in a water bath (Bactosonic, Bandelin, Berlin, Germany) at 40 kHz for 1 min. Then, the samples were thoroughly mixed, and MAC was determined by inoculating the test dilutions from each well on MH agar and incubated 24 h at 37 °C.

### 4.3. Biofilm Formation Assay

All tested *E. faecalis* strains were subcultured on MH agar for 18–24 h at 37 °C. From the grown bacterial colonies, a suspension was prepared in a physiological saline solution to an optical density of 0.08–1 corresponding to a density of 1.5 × 10^8^ bacteria/mL. These prepared bacterial suspensions were then inoculated into MH broth, and the optical density of the strain suspension was adjusted to a concentration of 1 × 10^6^ bacteria/mL. In a previously prepared series of test tubes containing dilutions of the tested antibiotics, aliquots (2.0 mL each) of the inoculated MH broth and a specific antibiotic dilution were mixed to achieve final antibiotic concentrations in the tubes of 1/2, 1/4, and 1/8 of the MIC for each strain. The fourth tube served as a control and contained only the bacterial suspension. In each well of a microtiter plate, 100 µL of the bacterial suspension and 100 µL of the respective antibiotic dilution were transferred, and the plates were incubated aerobically at 37 °C overnight (18–24 h). After incubation, the adhered cells were washed twice with PBS and treated in an ultrasonic bath (Bandelin-BactoSonic, Berlin, Germany) at 40 kHz for 1 min. Subsequently, the samples were thoroughly mixed, and a specified number of bacteria were plated by tenfold dilutions [76].

### 4.4. Examination of the Interaction of Selected Concentrations of Nitroxoline and Gentamicin on E. faecalis Strains Using the Checkerboard Assay

The checkerboard assay was employed to determine the interaction between NTX and GEN on selected uropathogenic *E. faecalis* strains. The highest concentrations of NTX and GEN were two times higher than the MIC. After determining the individual MIC values of NTX and GEN, their combination was tested on a microtiter plate. In plate 1, 50 µL of MH broth was added to all wells except in column 11, where 100 µL of the antibiotic working solution was added, followed by transferring 50 µL of the antibiotic into all wells except rows 1 and 2. In plate 2, 100 µL of MH broth was added to all rows except row G, where 200 µL of the GEN working solution was added, and then 100 µL of the GEN solution was transferred to all rows except rows A and B. Subsequently, 100 µL of bacterial suspension with a concentration of 1 × 10^6^ CFU/mL was added to all wells of plate 1. Plate 2 was used only for preparation. This process resulted in 36 different combinations of NTX and GEN concentrations. The GEN concentrations decreased by rows, from row G to row C, while the NTX concentrations decreased by columns, from column 11 to column 3. Column 2 was used as a GEN control, and row B was used as a NTX control. After 24 h of incubation at 35 ± 2 °C, the MIC values for the tested combination were determined based on turbidity within the wells.

### 4.5. Examination of the Bacterial Cell Morphology Using Transmission Electron Microscopy (TEM)

For the analysis of the impact of subinhibitory concentrations of NTX on *E. faecalis* cultures, the samples were prepared for electron microscope analysis to visualize potential structural and morphological changes. Ten µL of the bacterial suspension after treatment was applied onto copper grids coated with a Formvar layer (Agar Scientific Ltd, Stansted, Essex, United Kingdom) for 2 min. Excess liquid was removed from the grids using Whatman filter paper no. 3 (Macherey-Nagel, Düren, city, Germany). The remaining bacteria on the grids were stained with 1% phosphotungstic acid (Sigma-Aldrich, St. Louis, MO, USA) for 1 min. The grids were then left to air dry for several minutes. Prepared samples were examined using a transmission electron microscope (TEM) (JEM-2100F, Jeol, Tokyo, Japan).

### 4.6. Examination of the Protein Profile of Selected Uropathogenic Enterococci Using Matrix-Assisted Laser Desorption/Ionization Time-of-Flight Mass Spectrometry (MALDI-TOF MS)

The protocol recommended by the equipment manufacturer (extraction procedure, matrix, sample-matrix ratio) was adopted to ensure maximum compatibility with established procedures and easier implementation in the research experiments. For the direct identification method of colonies from nutrient agar, a thin film of bacteria was applied to a steel plate with 24 spots (Bruker Daltonics, Bremen, Germany) and allowed to dry at room temperature. After drying, 2.0 µL of MALDI matrix (saturated solution of α-cyano-4-hydroxycinnamic acid (HCCA; Bruker Daltonics, Bremen, Germany) in 50% acetonitrile and 2.5% trifluoroacetic acid) was applied to the colony and allowed to dry before testing [78]. The extraction method was performed according to Topić Popović et al. [79]. In this study, a pure bacterial suspension of *E. faecalis* with an optical density of 0.5 McFarland was used as a control and bacterial suspensions were treated with subinhibitory doses of NTX (8 µg/mL) and GEN (16 µg/mL).

Bacterial suspensions treated with a combination of NTX (8 µg/mL) and GEN (8 µg/mL) were also used. After 24 h of incubation, the suspensions were centrifuged at 15,000 rpm for 5 min, the supernatant was discarded, and the pellets were washed in a PBS solution. The sediment was carefully applied to a target plate, physiological saline was added to reduce sample density, and it was allowed to dry for a few minutes. Subsequently, 0.50 µL of 70% formic acid was added, allowed to dry, and finally, 1.00 µL of matrix (α-cyano-4-hydroxy-trans-cinnamic acid) was added and left to crystallize for a few minutes. Scoring criteria recommended by the manufacturer were used for identification: a score of ≥2000 indicated species-level identification, a score of 1700 to 1999 indicated genus-level identification, and a score <1700 was interpreted as unidentified [78]. The spectrum was analyzed and compared to the reference spectrum in the instrument’s database as well as with other control strains, selecting the "cleanest" spectrum without noise, with a score of at least ≥80. The obtained spectra were analyzed using the BioProfiler Expert program and the Flex Analysis program. Mass spectra of control strains and strains treated with antibiotics were compared to observe similarities and differences. Zero-line spectra and low signal-to-noise ratio spectra were discarded after visual inspection. Due to the large amount of data generated by these experiments, the mean spectrum for each isolate from instrumental and biological replicates was considered for further analysis. MS analyses were performed using the MALDI Biotyper Sirius® system (Bruker Daltonics, Bremen, Germany). Mass spectra were scanned in the range of *m*/*z* 3000 to 18000. Mass profiles were obtained using the FlexControl 3.4 software (Bruker Daltonics, Bremen, Germany). Spectra were recorded in linear positive ion mode (laser intensity 95%, ion source 1 = 10.00 kV, ion source 2 = 8.98 kV, lens = 3.00 kV, detector voltage = 2652 V, ion extraction pulse = 150 ns). Each spectrum corresponds to the accumulation of ions from 2000 to 5000 laser shots randomly divided. The obtained spectra were processed with default parameters using FlexAnalysis v.3.4 software (Bruker Daltonics, Bremen, Germany).

### 4.7. Statistical Analysis

All experiments were repeated three times, and the results are presented as the mean of three independent samples ± standard deviation (SD). Data processing was conducted using the computer program Statistica for Windows, version 12 (StatSoft Inc., Tulsa, OK, USA). Differences between data groups were analyzed using the non-parametric Mann–Whitney U test. Mean values between groups were compared using the Kruskal–Wallis ANOVA non-parametric test and the Mann–Whitney U test. Statistically significant values are considered when *p* < 0.05.

## 5. Conclusions

The results of this study demonstrate the synergistic effect of NTX and GEN for eight *E. faecalis* strains, including the control ATCC29212 strain, and the additive effect for the majority of the 29 tested strains. Keeping in mind that NTX does not show any pronounced side effects, that the rates of bacterial resistance towards NTX are exceptionally low, and that the resistance – if acquired – will be counteracted by a diminished virulence, a conclusion can be drawn that NTX deserves to be considered as the ampicillin replacement in the combined therapy with GEN for the treatment of UTIs. This could provide a substantial contribution to the ongoing fight against antimicrobial resistance and nosocomial infections.

However, due to the well-known nephrotoxicity of GEN, only the UTIs not requiring the prolonged use of drugs could be considered. It would thus be useful, alongside further research on the antiadhesion and antibiofilm effects of NTX, to dedicate part of the research efforts to finding alternatives for GEN in the treatment of systemic enterococcal infections. 

## Figures and Tables

**Table 1:** The values of minimum inhibitory concentrations (MICs) of gentamicin and nitroxoline, minimum bactericidal concentration (MBC), and minimum antiadhesion concentration (MAC) of nitroxoline for 29 *Enterococcus faecalis* strains. The strains are grouped based on the increase primarily of gentamicin MIC, then on the nitroxoline MIC.

*E. faecalis* Strain	Gentamicin	Nitroxoline			GROUP
	MIC (µg/mL)	MIC (µg/mL)	MBC (µg/mL)	MAC (µg/mL)	
E46	2	8	32	4	1
E61	4	16	>64	8	2
E34	8	2	32	2	3
E36	8	4	32	2	
ATCC29212	8	8	32	4	4
E69	8	8	>64	4	
E89	16	4	32	2	5
E85	16	4	32	2	
E10	16	4	32	2	
E16	16	8	>64	4	6
E35	16	8	>64	4	
E47	128	4	32	2	7
E15	128	4	32	2	
E32	128	4	32	2	
E84	128	4	32	2	
E95	128	4	32	2	
E79	128	8	32	4	8
E38	128	8	32	4	
E55	128	8	32	4	
E45	128	8	32	4	
E72	128	8	32	4	
E41	128	8	32	4	
E39	512	4	32	2	9
E8	512	4	32	2	
E58	512	4	32	2	
E1	512	4	32	2	
E7	512	8	32	4	10
E48	512	8	32	4	
E37	512	8	>64	4	

**Table 2:** *Enterococcus faecalis* strains and nitroxoline/gentamicin combinations with established synergistic antimicrobial effect.

Synergistic Antimicrobial Effect				
*E. faecalis* Strains	Individual MICs NTX/GEN (μg/mL)	Combination MICs NTX+GEN (μg/mL)		
GROUP 8 (E79, E38, E55, E45, E72, E41)	8/128	2/8	2/16	2/32
GROUP 4 (ATCC29212, E69)	8/8	2/0.5	2/1	2/2

MIC—Minimal inhibitory concentration; NTX—nitroxoline; GEN—gentamicin.

**Table 3:** *Enterococcus faecalis* strains and nitroxoline/gentamicin combinations with established additive antimicrobial effect.

Additive Antimicrobial Effect							
*E. faecalis* Strains	Individual MICs NTX/GEN (μg/mL)	Combination MICs NTX + GEN (μg/mL)					
GROUP 8 (E79, E38, E55, E45, E72, E41)	8/128	4/8	4/16	4/32	4/64	2/64	1/64
GROUP 4 (ATCC29212, E69)	8/8	4/0.5	4/1	4/2	4/4	2/4	
GROUP 7 (E47, E15, E32, E84, E95)	4/128	2/8	2/16	2/32	2/64	1/64	
GROUP 3 (E34, E36)	4/8	2/0.5	2/1	2/2	2/4	1/4	
GROUP 5 (E89, E85, E10)	4/16	2/2	2/4	2/8	1/8		
GROUP 2 (E61)	8/4	4/0.5	4/1	4/2			
GROUP 6 (E16, E35)	8/16	4/2	4/4	4/8			
GROUP 9 (E39, E8, E58, E1)	4/512	2/8	2/16	2/32	2/64	2/128	2/256
GROUP 10 (E7, E48, E37)	8/512	4/8	4/16	4/32	4/64	4/128	4/256
GROUP 1 (E46)	8/2	4/0.5	4/1	2/1			

MIC—Minimal inhibitory concentration; NTX—nitroxoline; GEN—gentamicin.

## Data Availability

Data are contained within the article. The raw data supporting the conclusions of this article will be made available by the authors on request.
